# Mortality Prediction by Bedside Rectus Femoris Muscle Ultrasound for Sarcopenia Diagnosis in Liver Cirrhosis

**DOI:** 10.1002/ueg2.70114

**Published:** 2025-10-04

**Authors:** Sara De Monte, Philipp Altmann, Svenja Pichlmeier, Hans Benno Leicht, Sophia Stuhlreiter, Roswitha Brandl, Florian P. Reiter, Sigrid Hahn, Clemens Benoit, Andreas Geier, Mathias Plauth, Monika Rau

**Affiliations:** ^1^ Division of Hepatology Department of Internal Medicine II University Hospital Würzburg Würzburg Germany; ^2^ Department of Diagnostic and Interventional Radiology University Hospital Würzburg Würzburg Germany; ^3^ Department of Nutritional, Food and Consumer Sciences University of Applied Siences Fulda Fulda Germany; ^4^ Division of Pediatric Radiology Department of Radiology University Hospital Würzburg Würzburg Germany; ^5^ Department of Internal Medicine Dessau Community General Hospital Dessau‐Rosslau Germany

**Keywords:** chair rise test, liver cirrhosis, morbidity, mortality, muscle function, prediction, rectus femoris muscle, sarcopenia, ultrasound

## Abstract

**Background:**

Sarcopenia is common in patients with liver cirrhosis and is an independent predictor of morbidity and mortality. This prospective study assessed the performance of rectus femoris muscle (RFM) ultrasound in patients with liver cirrhosis to identify those at risk for sarcopenia as defined by the combination of low muscle mass and low muscle strength.

**Methods:**

84 patients with liver cirrhosis hospitalized at a tertiary center (05/22‐02/24) were included with 6‐month follow‐up. Within 24–48 h of admission hand grip strength, chair rise test (CRT), timed up and go (TUG), short physical performance battery (SPPB), rectus femoris muscle ultrasound, and bioelectrical impedance analysis (BIA) were assessed. Statistical analyses included receiver operating characteristic (ROC) curves, Kaplan–Meier estimates, Cox regression, and competing risk analyses.

**Results:**

Most (73.8%) patients had decompensated and mainly alcohol‐related liver cirrhosis. Thickness and cross‐sectional area of rectus femoris muscle (MT_RFM_/CSA_RFM_) were significantly (*p* < 0.01 each) lower in more advanced disease by Child‐Pugh (CP) stage, also when normalized for height^2^. MT_RFM_/height^2^ and CSA_RFM_/height^2^ demonstrated good predictive value for BIA‐derived low muscle mass (ASMI < 7/5.7 kg/m^2^) or low phase angle ≤ 4.9° (AUROC 0.727–0.770). Impaired physical performance, in terms of prolonged CRT and TUG test time was associated with reduced MT_RFM_ or CSA_RFM_ (*p* < 0.05 each), respectively. Higher muscle echogenicity correlated with poorer performance in TUG and SPPB. Low rectus femoris muscle mass was associated with shorter survival and sarcopenic (prolonged CRT and low MT_RFM_/height^2^) patients had a high 6‐month mortality risk (HR 7.188; 95% CI 2.249–22.978).

**Conclusion:**

Rectus femoris muscle ultrasound is a feasible bedside method for identifying patients with liver cirrhosis at risk of sarcopenia. Sarcopenia as diagnosed by prolonged CRT together with low RFM mass by ultrasound is an independent predictor for 6‐month mortality, highlighting the clinical utility of RFM ultrasound in diagnosing sarcopenia.

## Key Summary

- Summarize the established knowledge on this subject
  ◦ Sarcopenia, defined by loss of muscle mass and strength, is common among patients with advanced liver disease and is an independent risk factor for poor outcomes.
- What are the significant and/or new findings of this study?
  ◦ In this study, bedside ultrasound of the rectus femoris muscle (RFM) and prolonged chair rise test time effectively identified cirrhotic patients with sarcopenia, who were found to have significantly higher 6‐month mortality rates.
  ◦ Incorporating sarcopenia assessment into routine care for patients with advanced liver disease is crucial, with bedside ultrasound serving as a practical and accessible tool.

## Graphical abstract

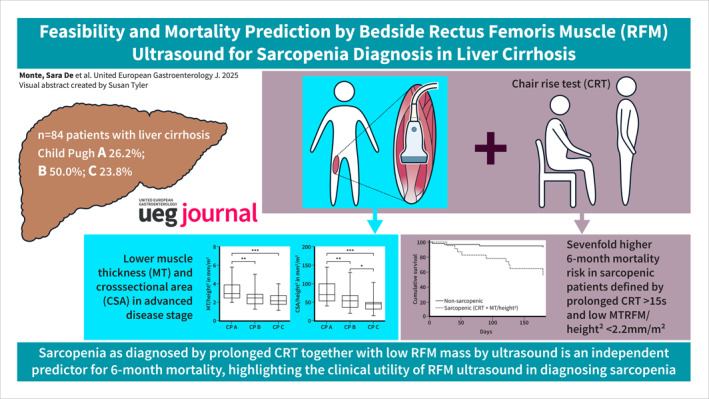

**Abbreviations**
ASMI: Appendicular skeletal muscle index
BIA: Bioelectrical impedance analysis
BSA: Body surface area
CP: Child–Pugh
CRT: Chair rise test
CSA: Cross‐sectional area
ECW: Extracellular water
EWGSOP: European Working Group on Sarcopenia in Older People
HGS: Hand grip strength
LC: Liver cirrhosis
MT: Muscle thickness
PhA: Phase angle
RFM: Rectus femoris muscle
ROC: Receiver operating characteristics
SGA: Subjective Global Assessment
SMI: Skeletal muscle mass index
SMM: Skeletal muscle mass
SPPB: Short physical performance battery
TBW: Total body water
TUG test: Timed Up and Go test

## 1 Introduction

Sarcopenia has emerged as an independent risk factor for morbidity and mortality in patients with chronic liver disease [1]. It is defined not only by low muscle mass but also by loss of muscle function, particularly low muscle strength [2]. Recent meta‐analyses reported an overall prevalence of sarcopenia in liver cirrhosis (LC) of 37.5% and 40.1%, respectively [3, 4], with higher rates observed in men, patients with alcohol‐related liver disease, and those with more severe disease. Notably, sarcopenia was associated with a 2.35‐fold higher mortality risk in patients with LC [3].

In 2019, the European Working Group on Sarcopenia in Older People (EWGSOP) updated the European consensus on sarcopenia diagnosis [2]. The current algorithm involves a positive screening followed by muscle strength assessment and confirmation by measuring muscle quantity or quality via bioelectrical impedance analysis (BIA) or imaging techniques such as MRI or CT [2].

In patients with LC, the assessment of muscle mass using abdominal CT or MRI images at the level of the third lumbar vertebra has become widely accepted. A low skeletal muscle mass index (SMI) is associated with higher mortality and is an independent predictor of first or further decompensation and of acute‐on‐chronic liver failure (ACLF) in patients with advanced chronic liver disease [5, 6, 7, 8, 9]. Accordingly, current guidelines recommend sarcopenia assessment in patients with LC to enable early identification of high‐risk patients, given its prognostic value independent of disease stage [1, 10].

Ultrasound has gained increasing interest as a reliable, affordable and portable method for assessing muscle mass at the bedside [11]. It has been shown that ultrasound strongly correlates with MRI‐, CT‐, and DXA‐based muscle measurements [11]. In ICU patients, rectus femoris muscle (RFM) cross‐sectional area (CSA) assessed by ultrasound compared with BIA‐derived phase angle (PhA) or skeletal muscle mass (SMM) was the only independent predictor of 28‐day‐mortality [12]. A recent study in patients with LC using RFM ultrasound found that a reduction in RFM mass was an independent predictor of acute decompensation within 1 year [13].

It should be noted, however, that in most studies using CT‐, MRI‐, or ultrasound‐based imaging, only low muscle mass was evaluated without assessing muscle strength and function, which are essential for the diagnosis of sarcopenia [2].

Given the importance of sarcopenia evaluation in predicting outcomes for patients with advanced liver disease, there is a need for a quick and reliable assessment tool that can be easily implemented at the bedside. Therefore, our study investigated the feasibility of bedside ultrasound for assessing low muscle mass as a component of sarcopenia diagnosis in hospitalized patients with advanced liver disease. Additionally, we systematically assessed muscle strength and physical performance to evaluate the second component of sarcopenia diagnosis.

## 2 Methods

### 2.1 Study Population

All patients with LC admitted to the hepatology ward of Würzburg University hospital between May 2022 and February 2024 were screened for study participation. LC was diagnosed by imaging (abdominal CT scan or liver‐MRI) and/or transient elastography (FibroScan). Patients with LC, regardless of etiology, were included within 48 h following hospital admission after informed consent was obtained. Exclusion criteria were acute liver failure, hepatocellular carcinoma, pregnancy, presence of a pacemaker, inability to stand on the BIA device, and inability to provide informed consent. Four patients with recent TIPS implantation were included, all of whom showed ongoing decompensation with ascites and hepatic encephalopathy during the study period despite the procedure. Six patients received TIPS implantation during the follow‐up period after study inclusion for secondary prophylaxis of bleeding and/or ascites. The study protocol was approved by Würzburg University’s Human Research Ethics Committee (AZ 11/22), registered (DRKS00035613) and conducted according to the guidelines of the Declaration of Helsinki. Follow‐up data were collected 6 months after patient enrollment, either during clinical follow‐up visits at our institution or through telephone interviews with the patients or their primary care physicians, if the patients were deceased.

### 2.2 Nutritional Status and Sarcopenia

At study inclusion, anthropometric data were collected, including weight, height, and circumferences of waist, hip, arm, thigh, and calf. Nutritional status was assessed using the Subjective Global Assessment (SGA) [14]. Hand grip strength (HGS) of the dominant hand was measured with the patient sitting on a hydraulic hand dynamometer (Baseline LiTE). Physical performance was evaluated using the short physical performance battery (SPPB) [15], which includes the balance test, 4‐m walking test, and chair rise test (CRT). In addition, the timed up and go (TUG) test was performed [16].

Cut‐offs for muscle strength and performance tests were applied according to EWGSOP2 [2] as follows: HGS < 16 kg for ♀/< 27 kg for ♂, CRT > 15 s for five chair rises, and SPPB score ≤ 8 as reported for patients with cirrhosis [17]. A TUG test ≥ 14 s was classified as prolonged based on previous data in patients with LC [18]. Routine laboratory blood tests were available for each patient.

### 2.3 Bioelectrical Impedance Analysis

Monofrequency (50 kHz) bioelectrical impedance analysis (BIA) was performed using a phase‐sensitive eight‐electrode device (seca mBCA 515; seca, Hamburg, Germany) according to a standard protocol [19]. Precision for resistance and reactance was evaluated by calculating the coefficient of variation for 15 BIA measurements taken in 1 day in a healthy subject. The coefficient of variation was 0.54% for resistance and 0.58% for reactance, respectively. In patients with ascites, BIA was performed within 12 h after paracentesis. The PhA cut‐off (≤ 4.9°) was chosen as published for patients with LC [19, 20]. Appendicular skeletal muscle index (ASMI), skeletal muscle mass (SMM), extracellular water (ECW) and total body water (TBW) were provided by the manufacturer's algorithm based on healthy controls [21, 22]. SMM and visceral adipose tissue (VAT) were validated against whole‐body MRI [21]. ASMI was calculated and cut‐offs (< 7 for males and < 5.7 for females) were used according to GLIM criteria [23]. Skeletal muscle mass index (SMI) was calculated based on SMM/m^2^ and gender‐specific cut‐offs were applied [24].

### 2.4 Standardized Ultrasound Examination

B‐mode ultrasound measurements were performed by two trained physicians using a linear probe (12–4 MHz; Philips Sparq; Philips Healthcare, Germany). Patients were positioned lying supine in a relaxed position with both knees extended and toes pointing upwards. Measurements were made at one fourth of the distance between the upper pole of the patella and the superior anterior iliac spine at the dominant leg. The probe was placed perpendicular to the longitudinal axis of the RFM with minimal pressure on the thigh soft tissue. Muscle thickness (MT_RFM_) and cross‐sectional area (CSA_RFM_) were measured in triplicates to calculate means for further analysis. Representative ultrasound images for MT_RFM_ and CSA_RFM_ are shown in Supporting Information S1: Figure S1. Interobserver agreement for the measurement of MT_RFM_ was assessed in a sub‐cohort of 17 patients, showing an excellent intraclass correlation coefficient (ICC) of 0.983 (CI 0.953–0.994). Muscle echogenicity was determined by gray‐scale histogram analysis of the images with ImageJ software (version 1.54d, National Institutes of Health, Bethesda, MD). The coefficient of variation for echogenicity was 3.9%, based on 15 measurements of RFM echogenicity in a single patient, reflecting high consistency. Echogenicity levels are expressed as grayscale values (0–255, unitless values).

### 2.5 Statistical Analysis

The sample size calculation was based on the comparison of sensitivity to diagnose low muscle mass by RFM ultrasound or ASMI by BIA with a difference of sensitivity of 50% (H0‐Hypothesis) and 75% (H1‐Hypothesis). The level of statistical power was set to 90% with a significance level (*α*) of 5%, resulting in a required sample size of 84 subjects. Sample size calculation is based on a two‐sided Binominal test using PASS 2020. Statistical analyses were performed using SPSS Statistics Version 27 (IBM SPSS; Chicago, IL, USA) and STATA version 19 (Stata Corp Lp., Texas, USA). Descriptive statistics were used to characterize the patient cohort. Normal distribution was assessed using Kolmogorov–Smirnov test. Univariate analyses were performed using Mann–Whitney *U*‐Test or *t*‐Test according to the distribution of data. Receiver operating characteristics (ROC) curves were generated to analyze the discriminatory capacity of ultrasound measures for ASMI < 7/5.7 kg/m^2^, PhA ≤ 4.9° and to assess the predictive value of CRT and ultrasound measures for 6‐month mortality. The Youden Index was used to determine the optimal cut‐off values for MT/height^2^ and CSA/height^2^ for predicting 6‐month mortality. Survival analyses for 6‐month mortality were performed using Kaplan–Meier curves and differences in the curves were compared using the log‐rank test. Time at risk was defined as time from study inclusion to death (including liver‐related death and non‐liver‐related death), liver transplant, or end of the follow‐up (180 days). Cox regression analyses were used for univariate and multivariate risk factor analyses. Patients were classified as “sarcopenic” based on the combination of low muscle strength, defined by CRT > 15 s, and low muscle mass, defined by CSA_RFM_/height^2^ < 51 mm^2^/mm^2^ or MT_RFM_/height^2^ < 2.2 mm/m^2^. *p* values < 0.05 were considered statistically significant. Competing risk analyses using the Fine and Gray method were performed to predict liver‐related death, with non‐liver‐related death or liver transplantation considered as competing risks, in sarcopenic and non‐sarcopenic patients. The results were visualized using cumulative incidence functions.

The primary endpoint was to assess the feasibility of RFM ultrasound for diagnosing low muscle mass in patients with advanced liver disease using BIA‐derived ASMI as a reference. Secondary endpoints included the analysis of RFM muscle parameters in predicting 6‐month mortality and identifying “sarcopenic” patients based on low muscle mass and low muscle strength.

## 3 Results

### 3.1 Cohort Demographics

**FIGURE 1:**
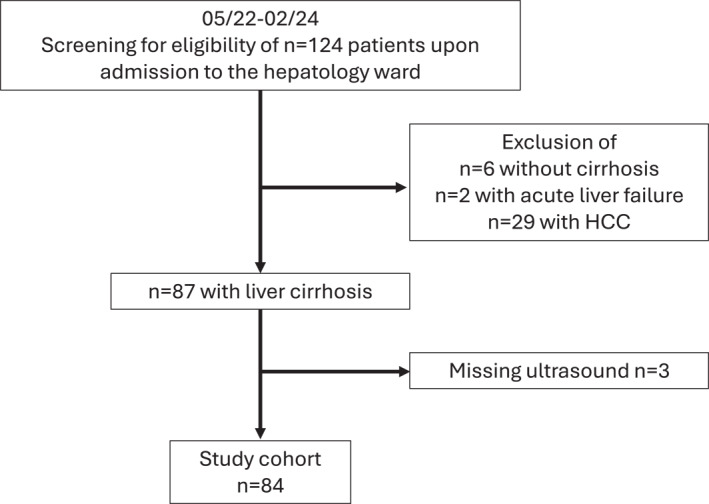
Flow diagram of study inclusion.

A total of 124 consecutive patients were screened for eligibility during the study period. Thirty seven patients did not meet inclusion criteria due to absence of cirrhosis, HCC, or acute liver failure (ALF). Eighty seven patients were included of whom three were excluded from analyses due to missing RFM ultrasound (see Figure 1 CONSORT flow diagram).

Ultimately, 84 patients (median age 60 years; IQR [52; 66]; 40% female) with a median MELD score of 16 [IQR 10; 20] were analyzed. Of these, 26.2% had compensated LC (Child–Pugh [CP] A), while the majority had decompensated LC (50.0% CP B; 23.8% CP C). Etiology of LC was mainly alcohol‐related liver disease (including two cases of MetALD) (48.8%), MASLD (15.5%), cholestatic liver disease (13.1%), viral hepatitis (7.1%), and other causes in 15.5%. Detailed cohort characteristics including clinical and laboratory parameters are shown in Table 1.

**TABLE 1:** Characteristics of the cohort.

Clinical data	Total *n* = 84
Sex (*m*/*w*)	60% (50)/40% (34)
Age (median [IQR])	60 [52; 68]
Etiology	
ALD/MASLD/cholestatic liver disease/viral/other % (*n*)	48.8% (41)/15.5% (13)/13.1% (11)/7.1% (6)/15.5% (13)
CP Stage (A/B/C) % (*n*)	26.2(22)/50.0(42)/23.8(20)
Decompensation episodes 1/≥ 2% (*n*)	29.8(25)/44.0(37)
Predominant decompensation	
Ascites % (*n*)	47.6 (40)
Jaundice % (*n*)	13.1 (11)
HE % (*n*)	9.5 (8)
Variceal bleeding % (*n*)	3.6 (3)
Medication	
ß‐blocker use % (*n*)	58.3 (49)
Antibiotic prophylaxis % (*n*)	10.7 (9)
Rifaximin use % (*n*)	29.8 (25)
Laboratory parameters	
Bilirubin mg/dL (median [IQR])	2.6 [1.0; 5.0]
Creatinine mg/dL (median [IQR])	1.0 [0.8; 1.4]
INR (median [IQR])	1.3 [1.1; 1.6]
WBC 10³/μL (median [IQR])	5.5 [3.5; 7.2]
Platelets 10³/μL (median [IQR])	93 [61; 134]
Albumin g/L (median [IQR])	35 [30; 41]
AST U/L (median [IQR])	50 [37; 66]
ALT U/L (median [IQR])	28 [18; 43]

### 3.2 Lower Muscle Thickness and Cross‐Sectional Area of RFM

**FIGURE 2:**
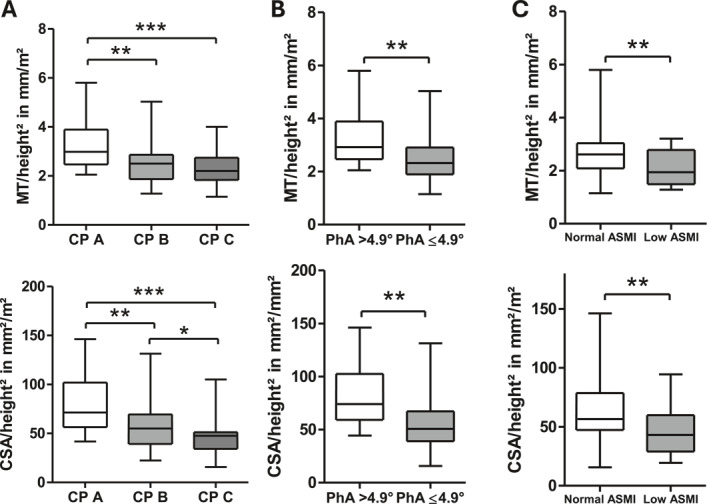
MT_RFM_ and CSA normalized for height^2^ across different CP stages (Panel A). MT_RFM_/height^2^ and CSA_RFM_/height^2^ according to normal (≥ 7/5.7 kg/m^2^) or low ASMI (< 7/5.7 kg/m^2^; Panel B) and PhA > 4.9° or ≤ 4.9° (Panel C). ***p < 0.001; **p < 0.01; *p < 0.05.

MT_RFM_ of patients with CP B (median 7.2 mm [IQR 5.8; 8.4]) and CP C (6.4 mm [6.0; 8.3]) was significantly (*p* < 0.01 each) lower than that of patients with CP A (8.3 mm [7.1; 11.1]; Supporting Information S1: Figure S2A). The same pattern was observed when normalizing MT_RFM_ for height, with MT_RFM_/height^2^ being significantly lower in patients with more severe disease (CP C vs. CP A (2.2 mm/m^2^ [1.8; 2.7]) versus 3.0 mm/m^2^ [2.5; 3.9]; *p* < 0.001) as well as in CP B versus CP A (2.5 mm/m^2^ [1.9; 2.9] vs. 3.0 mm/m^2^ [2.5; 3.9]; *p* < 0.01) (Figure 2A.) Similarly, the cross‐sectional area CSA_RFM_ was significantly lower in CP C versus CP A (137.3 mm^2^ [106.6; 161.7] vs. 204.9 mm^2^ [162.4; 287.8]; *p* < 0.001) as well as in CP B versus CP A (163.3 mm^2^ [121.4; 207.9] vs. 204.9 mm^2^ [163.0; 264.6]; *p* < 0.05) as shown in Supporting Information S1: Figure S2A. Likewise, CSA normalized for height (Figure 2A), was lowest in CP C compared to both CP A and CP B (CP C: 47.6 mm^2^/m^2^ [34.2:51.3] vs. CP A: 71.4 mm^2^/m^2^ [56.4; 101.9]; *p* < 0.001; CP C vs. CP B: 55.0 mm^2^/m^2^ [39.2; 69.4]; *p* < 0.05). Additionally, CSA_RFM_/height^2^ was significantly lower in CP B than in CP A (55.0 mm^2^/m^2^ [39.2; 69.4] vs. 71.4 mm^2^/m^2^ [56.4; 101.9]; *p* < 0.01). Normalizing MT and CSA for BSA yielded similar results (Supporting Information S1: Figure S2B). MT_RFM_/height^2^ and CSA_RFM_/height^2^ were significantly (*p* < 0.01) lower in LC patients with low ASMI or low PhA (≤ 4.9°) (Figure 2B,C).

**FIGURE 3:**
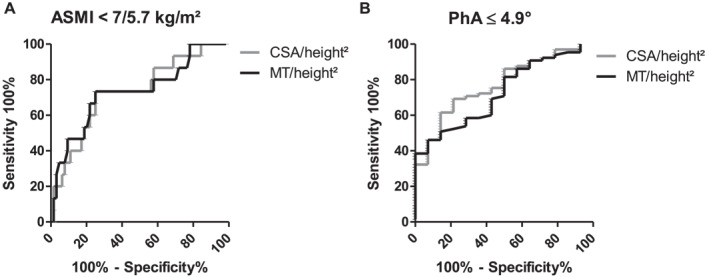
ROC of MT_RFM_/height^2^ and CSA_RFM_/height^2^ for (A) ASMI < 7/5.7 kg/m^2^ (males/females) and for (B) PhA ≤ 4.9°.

Identical AUROC values qualify MT_RFM_/height^2^ (0.730, CI 95% 0.577–0.883) and CSA_RFM_/height^2^ (0.730, CI 95% 0.585–0.875) as fair predictors of low BIA‐derived ASMI, the primary endpoint of our study (Figure 3A). Also, AUROC values of MT_RFM_/height^2^ (0.727; CI 95% 0.599–0.856) as well as for CSA_RFM_/height^2^ (0.770; CI 95% 0.649–0.892) performed equally well in predicting low PhA (Figure 3B).

### 3.3 RFM Ultrasound Outperformed BIA‐Derived ASMI in Detecting Low Muscle Mass in Decompensated Cirrhosis

**FIGURE 4:**
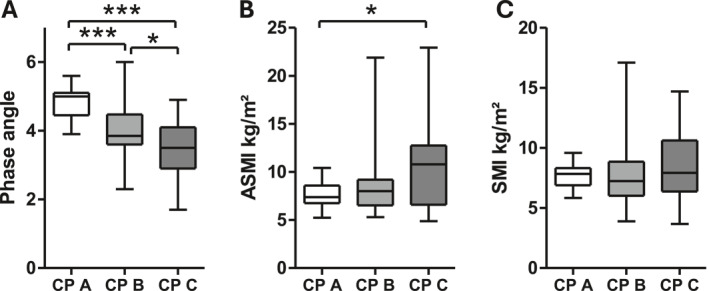
PhA according to CP stage (Panel A). ASMI and SMI according to CP stage (Panel B + C). *p < 0.05; ***p < 0.001.

In good accordance with ultrasound findings, raw data of BIA including PhA (Figure 4A) as well as reactance and resistance (Supporting Information S1: Figure S3) decreased significantly with more advanced disease. In sharp contrast, however, ASMI, SMI, and SMM as derived by regression equations from BIA measurements failed to detect this stage dependent decrease and even showed a tendency towards higher values in CP C patients (Figures 4B,C and S4). BIA‐derived estimates of extracellular or total body water showed higher values in patients with decompensated LC (Supporting Information S1: Figure S4).

### 3.4 Impaired Muscle Mass and Quality Are Associated With Decreased Muscle Strength and Performance

**FIGURE 5:**
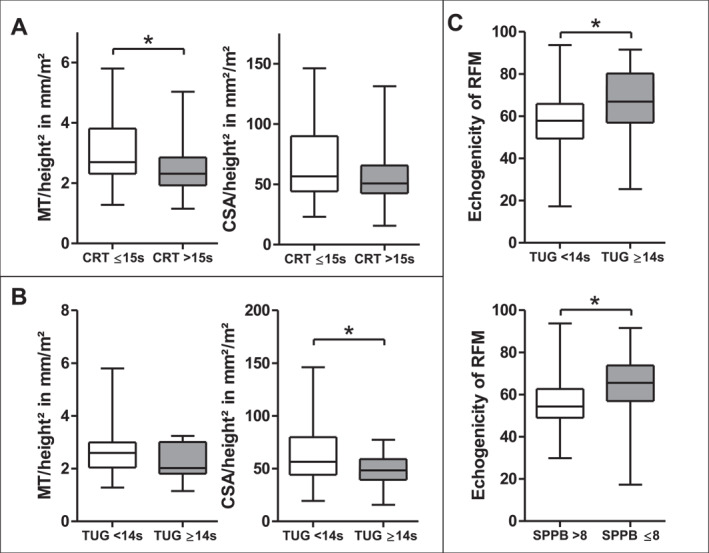
Low muscle thickness and cross‐sectional area by RFM in patients with low muscle strength in terms of prolonged CRT (A) and impaired physical performance in TUG (B). (C) Impaired muscle quality in terms of increased RFM echogenicity in frail patients. CRT = chair rise test, SPPB = short physical performance battery, TUG = timed up and go test. *p < 0.05.

In our patient cohort, 53/84 (63.1%) had a subnormal CRT, 24/84 (28.6%) had low HGS, and 17/84 (20.2%) had a prolonged TUG test time. In patients with a prolonged CRT time, MT_RFM_/height^2^ was significantly lower than in those with a normal CRT time (2.3 [1.9; 2.9] vs. 2.7 [2.3; 3.8] mm/m^2^; *p* < 0.05). Likewise, CSA_RFM_/height^2^ was significantly (*p* < 0.05) lower in patients with a prolonged TUG (Figure 5A,B). Normalization for body surface area showed similar results (Supporting Information S1: Figure S5). Low HGS showed no association with low MT_RFM_/height^2^ or low CSA_RFM_/height^2^ (Supporting Information S1: Figure S5).

Regarding muscle quality, patients with prolonged TUG time had higher echogenicity of RFM compared with those with normal TUG time (66.9 [56.9; 80.3] vs. 57.0 [49.2; 65.5] grayscale values; *p* < 0.05) (Figure 5C). Also, RFM echogenicity was significantly higher in frail patients (SPPB ≤ 8) compared to non‐frail patients (SPPB > 8; Figure 5C).

## 4 Sarcopenia, Combination of Low Muscle Mass and Strength, Predicts Mortality

After 6 months, 3/84 (3.6%) patients had received a liver transplant, and 13/84 (15.5%) patients had died. Mortality was liver‐related in 8/13 (61.5%) and non‐liver‐related in 5/13 (38.5%) of patients, the latter mainly due to cardiovascular events.

Predicting the composite 6‐month outcome death or transplantation, ROC analysis showed good AUC for CSA_RFM_/height^2^ 0.721 (CI 95% 0.590–0.851) and MT_RFM_/height^2^ 0.732 (CI 0.589–0.874) (Supporting Information S1: Figure S6A). Youden‐Index indicated cut‐offs for MT_RFM_/height^2^ at < 2.2 mm/mm^2^ and for CSA_RFM_/height^2^ at < 51 mm^2^/mm^2^, respectively. Kaplan–Meier analyses showed significant shorter survival in patients with low muscle mass by MT_RFM_/height^2^ < 2.2 mm/m^2^ (HR: 4.673 [CI 95% 1.621–13.472]) and by CSA_RFM_/height^2^ < 51 mm^2^/m^2^ (HR: 4.150 [CI 95% 1.336–12.884]; Supporting Information S1: Figure S7). In a sensitivity analysis excluding six patients who had a TIPS implanted during the follow‐up period, AUC for CSA_RFM_/height^2^ 0.724 (CI 95% 0.593–0.856) and MT_RFM_/height^2^ 0.738 (CI 0.596–0.879) remained unchanged.

**FIGURE 6:**
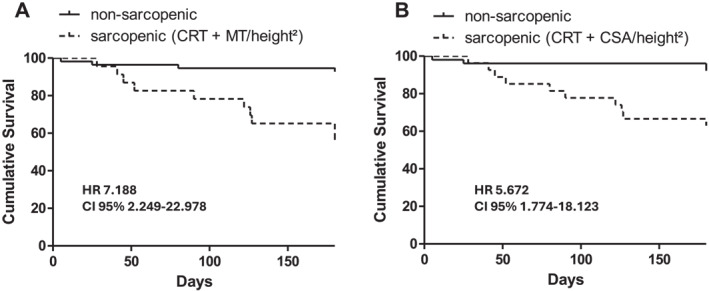
Survival analysis of patients classified as sarcopenic in terms of CRT > 15 s and MT/height^2^ < 2.2 mm/m^2^ (Panel A) and CRT > 15 s and CSA/height^2^ < 51 mm^2^/m^2^ (Panel B).

Since the diagnosis of sarcopenia requires not only low muscle mass but also low muscle strength, CRT and HGS were analyzed for predicting 6‐month mortality. CRT time showed a statistically significant AUC of 0.852 (CI 95% 0.749–0.954) for the prediction of 6‐month mortality (Supporting Information S1: Figure S6B), while that for HGS was not significant (data not shown). Patients classified as sarcopenic by the combination of prolonged CRT time and either low MT_RFM_/height^2^ (HR 7.188; CI 95% 2.249–22.978) or low CSA_RFM_/height^2^ (HR 5.672; CI 95% 1.774–18.123) had a significantly higher mortality risk compared to non‐sarcopenic patients (Figure 6). Even higher HR was obtained when MT_RFM_ or CSA_RFM_ were normalized for body surface area (Table 2 and Supporting Information S1: Figure S8).

**TABLE 2:** Univariate and multivariate cox regression analysis for mortality.

	*p* value	HR (CI 95%)
Univariate analysis		
Age	0.432	1.020 (0.970–1.073)
BMI	0.876	1.007 (0.924–1.098)
MELD	**< 0.001**	1.165 (1.088–1.247)
INR	**0.002**	4.545 (1.711–12.071)
Bilirubin	**0.016**	1.063 (1.010–1.118)
Creatinine	**< 0.001**	3.897 (1.900–7.994)
PhA	**< 0.001**	0.355 (0.202–47.128)
ASMI	0.284	1.071 (0.945–1.213)
Low CSA/height²	**0.014**	4.150 (1.336–12.884)
Sarcopenic (CRT + CSA/height²)	**0.003**	5.672 (1.774–18.123)
Low MT/height²	**0.004**	4.673 (1.621–13.472)
Sarcopenic (CRT + MT/height²)	**0.001**	7.188 (2.249–22.978)
Low CSA/BSA	**0.004**	5.180 (1.667–16.090)
Sarcopenic (CRT + CSA/BSA)	**0.001**	7.509 (2.346–24.039)
Low MT/BSA	**0.001**	5.647 (1.957–16.290)
Sarcopenic (CRT + MT/BSA)	**< 0.001**	9.892 (3.089–31.679)
Multivariate analysis		
Sarcopenic (CRT + MT/height²)	**0.019**	4.348 (1.268–14.903)
MELD	**0.001**	1.127 (1.050–1.210)

*Note:* The bold values are statistically significant values.

In univariate risk factor analyses for 6‐month mortality (Table 2), all RFM ultrasound measurements and sarcopenia classifications based on CRT + RFM ultrasound were identified as risk factors, along with MELD, INR, bilirubin, creatinine, and PhA. Furthermore, in multivariate analysis, sarcopenia based on CRT + MT_RFM_/height^2^ was found to be an independent predictor of mortality as well as MELD score (HR for CRT + MT_RFM_/height^2^ 4.348 [CI 95% 1.268–14.903]; Table 2).

Competing risk analysis for liver‐related death considering non‐liver‐related death (*n* = 5) or liver transplantation (*n* = 3) as competing events showed that sarcopenic patients defined by either CRT + MT/height^2^ or CRT + CSA/height^2^ were at increased risk of liver‐related death (Supporting Information S1: Figures S9 and S10).

## 5 Discussion

This prospective study demonstrates the feasibility of using RFM ultrasound to assess low muscle mass in patients with mainly decompensated LC and highlights the prognostic value of bedside ultrasound‐derived measures of muscle mass in predicting 6‐month mortality. The risk of 6‐month mortality was significantly higher when sarcopenia was defined by the combination of low RFM muscle mass and low muscle strength.

Our study showed that muscle mass assessment by RFM ultrasound was feasible and both MT_RFM_ as well as CSA_RFM_ were significantly lower in patients with LC in a stage dependent manner, also when normalized for height^2^ or BSA. Furthermore, low RFM mass by ultrasound showed a significant association with low BIA‐derived PhA or ASMI. Both MT_RFM_/height^2^ and CSA_RFM_/height^2^ demonstrated good predictive value for low PhA and low ASMI, with AUROC ranging from 0.730 to 0.770. In patients with LC (60% CP A), Tandon et al. showed that the combination of full thigh muscle thickness measured by ultrasound and body mass index (BMI) identified sarcopenia in both male and female patients almost as effectively as CT‐ or MRI‐based SMI with AUROC values (women: 0.78; men: 0.89) comparable to our study [25]. Notably, approach of focusing on measuring RFM mass in our study offered the advantage of obtaining measurements even in patients with decompensated cirrhosis and profound hypervolemia in whom the assessment of the entire thigh muscle would have been more challenging.

Our analyses confirmed the limitations of BIA‐derived ASMI and SMI in the assessment of muscle mass in patients with LC. While PhA was significantly reduced in patients with CP B or C, ASMI and SMI were not and showed high variability, particularly in the CP C stage. Similar limitations of BIA‐derived SMI and SMM have been noted in a prospective study of 106 patients with LC [26]. In those with severe decompensation, a weaker correlation was observed between BIA‐derived SMI or SMM and CT‐derived SMI (L3), whereas the correlation between PhA and CT‐based SMI was more robust. Equations used to calculate TBW, SMI, or SMM from BIA raw values (resistance and reactance) are typically population‐ and device‐specific. Therefore, they are only applicable to individuals who share the characteristics of the reference population [27]. Importantly, PhA has been shown to be a reliable tool for diagnosing sarcopenia regardless of the presence of ascites in patients with LC [28] and to be a stronger predictor of hospital outcome than SGA in hospitalized patients [29]. Also, in patients with cirrhosis on the waiting list for transplantation, only three out of 36 different combinations of phenotypic and etiologic GLIM criteria independently predicted mortality that used low PhA as an indicator of reduced muscle mass [30]. In our study, PhA proved superior to BIA‐derived ASMI in the detection of muscle mass even in patients with decompensated LC.

Furthermore, we observed that prolonged CRT and TUG test time, reflecting lower limb strength and performance but not HGS, were strongly associated with reduced RFM mass. Corroborating our observation, a greater impairment in lower limb as compared to arm and trunk muscle function has been observed in patients with LC by other investigators [31]. Moreover, we found a significant association between poor muscle quality in terms of increased RFM‐echogenicity, indicative of myosteatosis, and both prolonged TUG test time and reduced SPPB performance. This is in good agreement with findings in 50 patients with LC and minimal hepatic encephalopathy showing an association between TUG test time and myosteatosis as assessed by CT and an increased rate of falls in patients with a prolonged TUG test [18]. These findings suggest a link between altered muscle quality and impaired physical performance, warranting further investigation into the underlying mechanisms. By measuring echogenicity, ultrasound is valuable for assessing not only muscle mass but also quality, which is crucial given the independent risk of myosteatosis, alone or with sarcopenia, for poor outcomes in patients with LC [32].

Sarcopenia in terms of low muscle mass in combination with impaired muscle strength and also low muscle mass alone were independent risk factors for 6‐month mortality in our patient cohort with predominantly decompensated LC. RFM ultrasound findings demonstrated good predictive power for 6‐month mortality and Kaplan–Meier analyses revealed significantly reduced survival in patients with low RFM mass. This is in good agreement with findings of a study in 63 patients with LC without TIPS showing that RFM ultrasound was predictive of acute decompensation within 1 year and was comparable to CT‐based SMI; with moderate agreement between CT and US was moderate [13]. In this study, however, muscle strength was not assessed and there was greater variability in ultrasound measurements, most likely due to the number of operators. While most studies in LC have focused on either CT‐based SMI to assess low muscle mass [6, 33] or frailty to assess impaired muscle function [34] as predictors of poor outcome, we combined the assessment of low RFM mass and low muscle function assessed by CRT to diagnose sarcopenia according to EWGSOP2 criteria [2]. Sarcopenic (CRT > 15 s and MT/height^2^ < 2.2 mm/m^2^) patients had a sevenfold higher 6‐month mortality risk (HR 7.188) compared to non‐sarcopenic patients. Using this combination in multivariate analysis, sarcopenia was an independent risk factor for mortality along with MELD. Competing risk analysis yielded a significantly higher risk of 6‐month liver‐related death in sarcopenic versus non‐sarcopenic patients, considering non‐liver related death or liver transplantation as competing events using the combination CRT + MT/height^2^ or CRT + CSA/height^2^ to diagnose sarcopenia. These results, however, should be interpreted cautiously due to the small number of competing events.

Taken together, our data demonstrate that the combination of both CRT and RFM ultrasound can reliably identify patients at high risk of adverse clinical outcomes. Importantly, such assessment can be performed quickly and easily at the bedside, making CRT and RFM ultrasound strong candidates for diagnosing sarcopenia in routine clinical practice.

Limitations of our study include the fact that patients were drawn from a single tertiary referral center, which may limit their generalizability. Additionally, the sample size was not used to analyze sex‐specific differences in the assessment of muscle mass using ultrasound of RFM; therefore, we cannot fully exclude sex‐related bias. Third, only 26% of patients had compensated liver cirrhosis according to CP stage, and this subgroup should be investigated in future studies. The high proportion of patients with decompensated LC, however, adds strength to our findings as, due to higher risk, they are in greater need for valid assessment of sarcopenia. Furthermore, we showed that the accuracy of BIA‐derived ASMI is limited in these patients. Fourth, the limited number of competing events requires cautious interpretation of competing risk analyses. Lastly, in immobilized patients who were unable to stand on the BIA device and who could not perform the CRT, our proposed diagnostic approach for sarcopenia is not applicable to this severely ill subgroup. Further studies are needed to evaluate whether bedside ultrasound assessment of RFM alone could serve as a substitute in such cases.

In conclusion, our study demonstrated the feasibility of using RFM ultrasound to detect low muscle mass in patients with LC even in states of decompensation. The combination of RFM ultrasound and CRT proved to be a practical and effective tool for identifying patients with sarcopenia. In patients with hepatic cirrhosis, low RFM mass and sarcopenia as diagnosed by both low RFM mass and impaired muscle function identify patients with an up to 7‐fold higher mortality risk. Future studies are needed to generate age‐ and sex‐specific cut‐off values for RFM muscle mass and quality. Given the increasing prevalence of obesity and sarcopenic obesity in patients with LC, RFM ultrasound may also serve as a valuable tool for assessing sarcopenia in such patients and this potential should be explored in future studies.

## Author Contributions

Conceptualization: Sara De Monte, Hans Benno Leicht, and Monika Rau. Provision of patients: Sara De Monte, Philipp Altmann, Roswitha Brandl, Sophia Stuhlreiter, and Monika Rau. Bioinformatic and statistical analyses: Philipp Altmann, and Monika Rau. Data analysis and interpretation: Sara De Monte, and Svenja Pichlmeier, Clemens Benoit, Sigrid Hahn, Mathias Plauth, and Monika Rau. Writing original draft preparation: Sara De Monte, Mathias Plauth, and Monika Rau. Writing – reviewing and editing: all authors

## Ethics Statement

The study was reviewed and approved by the Institutional Review Board (Ethics Committee of Würzburg, AZ11/22).

## Consent

All patients gave written informed consent before enrollment in the monocentric study cohort.

## Conflicts of Interest

The authors declare no conflicts of interest.

## Supporting information


Supporting Information S1

## Data Availability

The data that support the findings of this study are available from the corresponding author upon reasonable request.

## References

[1] M. Plauth , W. Bernal , S. Dasarathy , et al., “ESPEN Guideline on Clinical Nutrition in Liver Disease,” Clinical Nutrition 38, no. 2 (2019): 485–521, 10.1016/j.clnu.2018.12.022. 30712783 PMC6686849

[2] A. J. Cruz‐Jentoft , G. Bahat , J. Bauer , et al., “Sarcopenia: Revised European Consensus on Definition and Diagnosis,” Age and Ageing 48, no. 1 (2019): 16–31, 10.1093/ageing/afy169. 30312372 PMC6322506

[3] X. Tantai , Y. Liu , Y. H. Yeo , et al., “Effect of Sarcopenia on Survival in Patients With Cirrhosis: A Meta‐Analysis,” Journal of Hepatology 76, no. 3 (2022): 588–599, 10.1016/j.jhep.2021.11.006. 34785325

[4] S. Tuo , Y. H. Yeo , R. Chang , et al., “Prevalence of and Associated Factors for Sarcopenia in Patients With Liver Cirrhosis: A Systematic Review and Meta‐Analysis,” Clinical Nutrition 43, no. 1 (2024): 84–94, 10.1016/j.clnu.2023.11.008. 38016243

[5] A. Faron , J. Abu‐Omar , J. Chang , et al., “Combination of Fat‐Free Muscle Index and Total Spontaneous Portosystemic Shunt Area Identifies High‐Risk Cirrhosis Patients,” Frontiers of Medicine 9 (2022): 831005, 10.3389/fmed.2022.831005. PMC9040492 35492329

[6] A. J. Montano‐Loza , J. Meza‐Junco , C. M. Prado , et al., “Muscle Wasting Is Associated With Mortality in Patients With Cirrhosis,” Clinical Gastroenterology and Hepatology 10, no. 2 (2012): 166–173: 173.e161, 10.1016/j.cgh.2011.08.028. 21893129

[7] R. Paternostro , C. Bardach , B. S. Hofer , et al., “Prognostic Impact of Sarcopenia in Cirrhotic Patients Stratified by Different Severity of Portal Hypertension,” Liver International 41, no. 4 (2021): 799–809, 10.1111/liv.14758. 33290614 PMC8048669

[8] G. G. Perdiguero , J. C. Spina , J. Martínez , et al., “Enhancing ACLF Prediction by Integrating Sarcopenia Assessment and Frailty in Liver Transplant Candidates on the Waiting List,” JHEP Reports 6, no. 3 (2024): 100985, 10.1016/j.jhepr.2023.100985. 38384670 PMC10879792

[9] M. Praktiknjo , M. Book , J. Luetkens , et al., “Fat‐Free Muscle Mass in Magnetic Resonance Imaging Predicts Acute‐on‐Chronic Liver Failure and Survival in Decompensated Cirrhosis,” Hepatology 67, no. 3 (2018): 1014–1026, 10.1002/hep.29602. 29059469

[10] M. Merli , A. Berzigotti , S. Zelber‐Sagi , et al., “EASL Clinical Practice Guidelines on Nutrition in Chronic Liver Disease,” Journal of Hepatology 70, no. 1 (2019): 172–193, 10.1016/j.jhep.2018.06.024. 30144956 PMC6657019

[11] S. Perkisas , S. Bastijns , S. Baudry , et al., “Application of Ultrasound for Muscle Assessment in Sarcopenia: 2020 SARCUS Update,” European Geriatric Medicine Society 12, no. 1 (2021): 45–59, 10.1007/s41999-020-00433-9. 33387359

[12] L. B. da Silva Passos , T. A. A. Macedo , and D. A. De‐Souza , “Nutritional State Assessed by Ultrasonography, but Not by Bioelectric Impedance, Predicts 28‐Day Mortality in Critically Ill Patients. Prospective Cohort Study,” Clinical Nutrition 40, no. 12 (2021): 5742–5750, 10.1016/j.clnu.2021.10.015. 34763258

[13] J. Gödiker , L. Schwind , T. Jacob , et al., “Ultrasound‐Defined Sarcopenia Independently Predicts Acute Decompensation in Advanced Chronic Liver Disease,” Journal of Cachexia, Sarcopenia and Muscle 15, no. 6 (2024): 2792–2802, 10.1002/jcsm.13630. 39529225 PMC11634521

[14] T. P. M. Schütz , “Subjective Global Assessment—Eine Methode zur Erfassung des Ernährungszustandes,” Aktuelle Ernährungsmedizin 30 (2005): 43–48, 10.1055/s-2004-834559.

[15] J. M. Guralnik , E. M. Simonsick , L. Ferrucci , et al., “A Short Physical Performance Battery Assessing Lower Extremity Function: Association With Self‐Reported Disability and Prediction of Mortality and Nursing Home Admission,” Journal of Gerontology 49, no. 2 (1994): M85–M94, 10.1093/geronj/49.2.m85. 8126356

[16] D. Podsiadlo and S. Richardson , “The Timed ‘Up & Go’: A Test of Basic Functional Mobility for Frail Elderly Persons,” Journal of the American Geriatrics Society 39, no. 2 (1991): 142–148, 10.1111/j.1532-5415.1991.tb01616.x. 1991946

[17] J. C. Lai , S. Feng , N. A. Terrault , B. Lizaola , H. Hayssen , and K. Covinsky , “Frailty Predicts Waitlist Mortality in Liver Transplant Candidates,” American Journal of Transplantation 14, no. 8 (2014): 1870–1879, 10.1111/ajt.12762. 24935609 PMC4107151

[18] S. Nardelli , S. Gioia , L. Ridola , et al., “Risk of Falls in Patients With Cirrhosis Evaluated by Timed Up and Go Test: Does Muscle or Brain Matter More?,” Digestive and Liver Disease 54, no. 3 (2022): 371–377, 10.1016/j.dld.2021.06.019. 34233863

[19] G. Belarmino , M. C. Gonzalez , R. S. Torrinhas , et al., “Phase Angle Obtained by Bioelectrical Impedance Analysis Independently Predicts Mortality in Patients With Cirrhosis,” World Journal of Hepatology 9, no. 7 (2017): 401–408, 10.4254/wjh.v9.i7.401. 28321276 PMC5340995

[20] A. Ruiz‐Margáin , R. U. Macías‐Rodríguez , A. Duarte‐Rojo , S. L. Ríos‐Torres , Á Espinosa‐Cuevas , and A. Torre , “Malnutrition Assessed Through Phase Angle and Its Relation to Prognosis in Patients With Compensated Liver Cirrhosis: A Prospective Cohort Study,” Digestive and Liver Disease 47, no. 4 (2015): 309–314, 10.1016/j.dld.2014.12.015. 25618555

[21] A. Bosy‐Westphal , B. Jensen , W. Braun , M. Pourhassan , D. Gallagher , and M. J. Müller , “Quantification of Whole‐Body and Segmental Skeletal Muscle Mass Using Phase‐Sensitive 8‐Electrode Medical Bioelectrical Impedance Devices,” European Journal of Clinical Nutrition 71, no. 9 (2017): 1061–1067, 10.1038/ejcn.2017.27. 28327564 PMC5589975

[22] A. Bosy‐Westphal , B. Schautz , W. Later , J. J. Kehayias , D. Gallagher , and M. J. Müller , “What Makes a BIA Equation Unique? Validity of Eight‐Electrode Multifrequency BIA to Estimate Body Composition in a Healthy Adult Population,” supplement, European Journal of Clinical Nutrition 67, no. S1 (2013): S14–S21, 10.1038/ejcn.2012.160. 23299866

[23] T. Cederholm , G. L. Jensen , M. Correia , et al., “GLIM Criteria for the Diagnosis of Malnutrition—A Consensus Report From the Global Clinical Nutrition Community,” Clinical Nutrition 38 (2019): 1–9, 10.1002/jcsm.12383. 30181091

[24] C. O. Walowski , W. Braun , M. J. Maisch , et al., “Reference Values for Skeletal Muscle Mass—Current Concepts and Methodological Considerations,” Nutrients 12, no. 3 (2020): 755, 10.3390/nu12030755. 32178373 PMC7146130

[25] P. Tandon , G. Low , M. Mourtzakis , et al., “A Model to Identify Sarcopenia in Patients With Cirrhosis,” Clinical Gastroenterology and Hepatology 14, no. 10 (2016): 1473–1480.e1473, 10.1016/j.cgh.2016.04.040. 27189915

[26] D. Bozic , I. Grgurevic , B. Mamic , et al., “Detection of Sarcopenia in Patients With Liver Cirrhosis Using the Bioelectrical Impedance Analysis,” Nutrients 15 (2023): 3335, 10.3390/nu15153335. 37571273 PMC10421520

[27] M. Marra , R. Sammarco , A. De Lorenzo , et al., “Assessment of Body Composition in Health and Disease Using Bioelectrical Impedance Analysis (BIA) and Dual Energy X‐Ray Absorptiometry (DXA): A Critical Overview,” Contrast Media and Molecular Imaging 2019 (2019): 3548284–3548289, 10.1155/2019/3548284. 31275083 PMC6560329

[28] A. Ruiz‐Margáin , J. J. Xie , B. M. Román‐Calleja , et al., “Phase Angle From Bioelectrical Impedance for the Assessment of Sarcopenia in Cirrhosis With or Without Ascites,” Clinical Gastroenterology and Hepatology 19, no. 9 (2021): 1941–1949.e1942, 10.1016/j.cgh.2020.08.066. 32890753

[29] M. Plauth , I. Sulz , M. Viertel , et al., “Phase Angle Is a Stronger Predictor of Hospital Outcome Than Subjective Global Assessment‐Results From the Prospective Dessau Hospital Malnutrition Study,” Nutrients 14, no. 9 (2022): 1780, 10.3390/nu14091780. 35565747 PMC9100773

[30] B. C. Santos , A. L. F. Fonseca , L. G. Ferreira , et al., “Different Combinations of the GLIM Criteria for Patients Awaiting a Liver Transplant: Poor Performance for Malnutrition Diagnosis but a Potentially Useful Prognostic Tool,” Clinical Nutrition 41, no. 1 (2022): 97–104, 10.1016/j.clnu.2021.11.008. 34864459

[31] J. I. Quinlan , A. Dhaliwal , F. R. Williams , et al., “Impaired Lower Limb Muscle Mass, Quality and Function in End Stage Liver Disease: A Cross‐Sectional Study,” Experimental Physiology 108, no. 8 (2023): 1066–1079, 10.1113/ep091157. 37166422 PMC10988432

[32] S. Di Cola , G. D’Amico , P. Caraceni , et al., “Myosteatosis Is Closely Associated With Sarcopenia and Significantly Worse Outcomes in Patients With Cirrhosis,” Journal of Hepatology 81, no. 4 (2024): 641–650, 10.1016/j.jhep.2024.05.020. 38782120

[33] A. J. Montano‐Loza , A. Duarte‐Rojo , J. Meza‐Junco , et al., “Inclusion of Sarcopenia Within MELD (MELD‐Sarcopenia) and the Prediction of Mortality in Patients With Cirrhosis,” Clinical and Translational Gastroenterology 6, no. 7 (2015): e102, 10.1038/ctg.2015.31. 26181291 PMC4816259

[34] J. C. Lai , A. M. Shui , A. Duarte‐Rojo , et al., “Frailty, Mortality, and Health Care Utilization After Liver Transplantation: From the Multicenter Functional Assessment in Liver Transplantation (FrAILT) Study,” Hepatology 75, no. 6 (2022): 1471–1479, 10.1002/hep.32268. 34862808 PMC9117399

