# Supplementary material for: Mortality Prediction by Bedside Rectus Femoris Muscle Ultrasound for Sarcopenia Diagnosis in Liver Cirrhosis
Source: United European Gastroenterol J. 2025 Oct 4;13(10):1936–45. doi: 10.1002/ueg2.70114 (PMC12704570; doi:10.1002/ueg2.70114)
Supplement: Supplementary file 1 — Supporting Information S1 [file UEG2-13-1936-s001.docx]

**Supplemental Figures**:

**
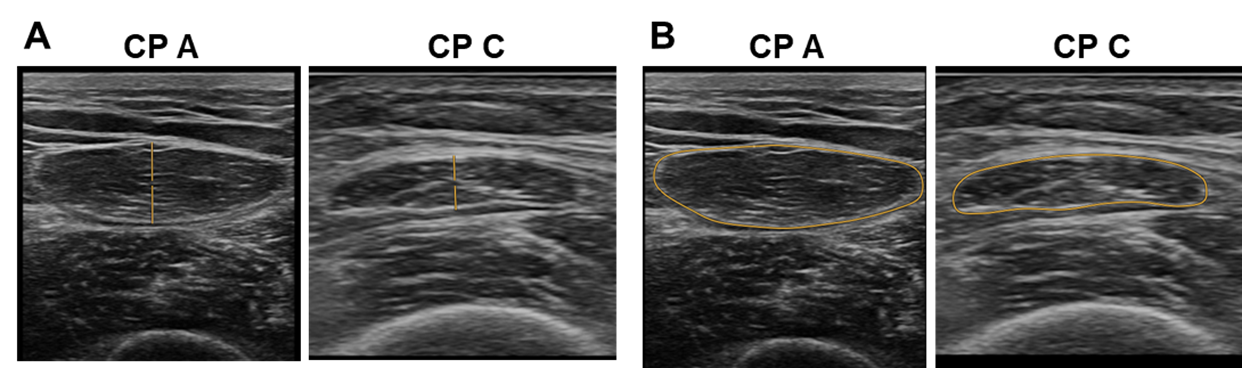
**

**Suppl. Fig. 1**: Representative ultrasound images for MT_RFM_ (**Panel A**) and CSA_RFM_ (**Panel B**).


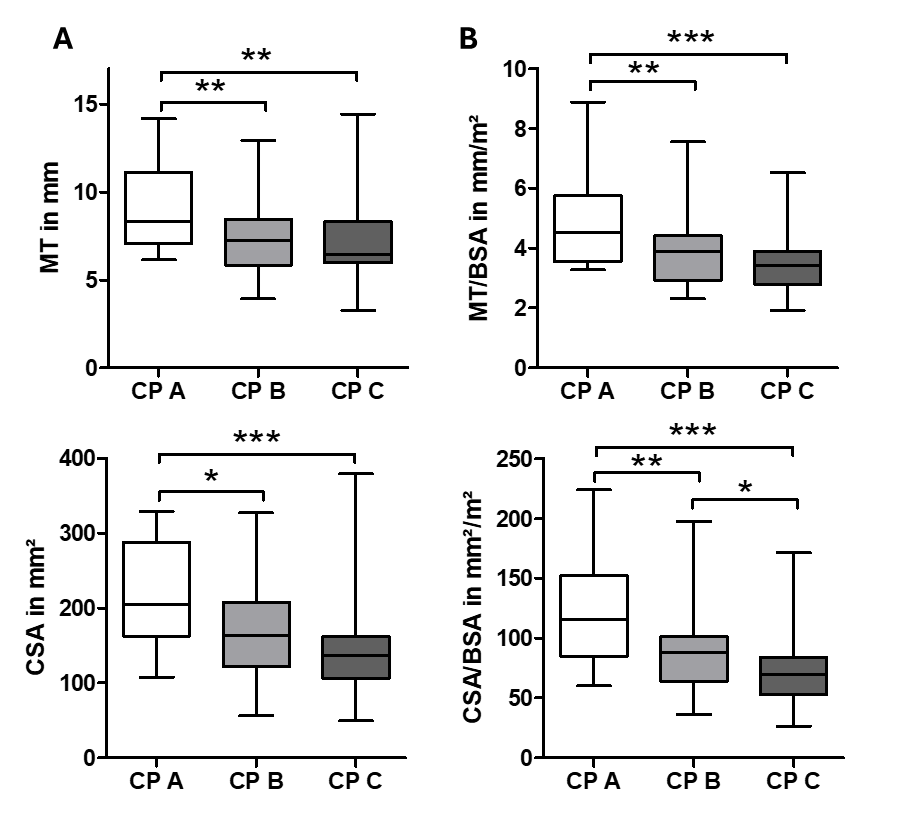


**Suppl. Fig. 2**: MT and CSA (**Panel A**) as well as MT_RFM_/BSA and CSA_RFM_/BSA (**Panel B**), across different CP stages. * = p<0.05; ** = p<0.01.

**
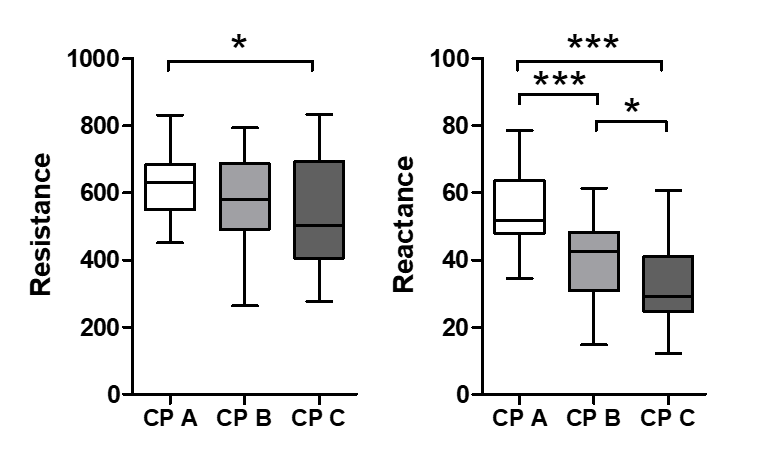
**

**Suppl. Fig. 3**: Differences of BIA raw data according to CP stage. * = p<0.05; ** = p<0.01; *** = p<0.001.

**
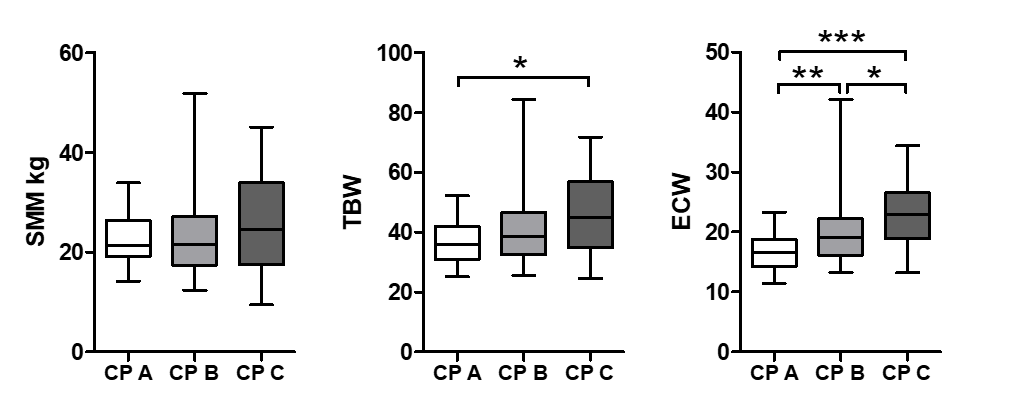
**

**Suppl. Fig. 4**: Differences of BIA parameters according to CP stage. * = p<0.05; ** = p<0.01; *** = p<0.001. Skeletal muscle mass (SMM), extracellular body water (EBW) or total body water (TBW).

**
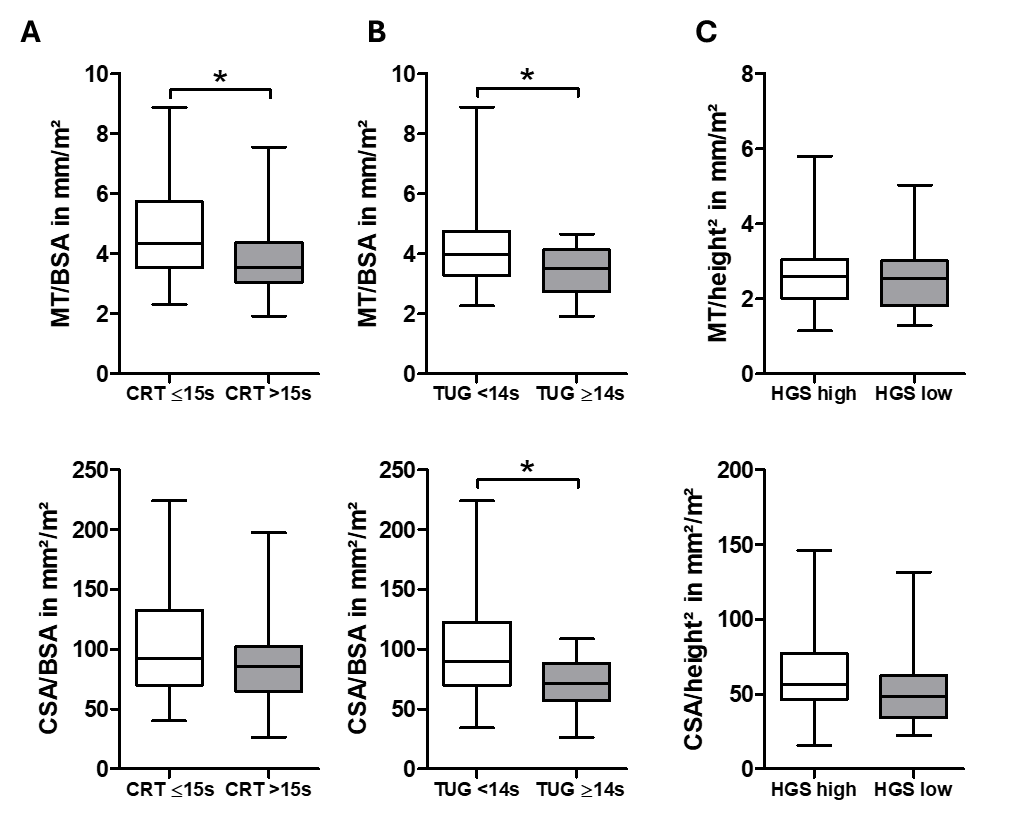
**

**Suppl. Fig. 5**: Differences of muscle mass of RFM based on muscle strength tests including CRT (**Panel A**) and physical performance in TUG (**Panel B**) for MT/BSA and CSA/BSA. MT/height² and CSA/height² in patients with high and low HGS (**Panel C**). CRT=chair rise test. HGS=hand grip strength, TUG=timed up and go test. * = p<0.05.

**
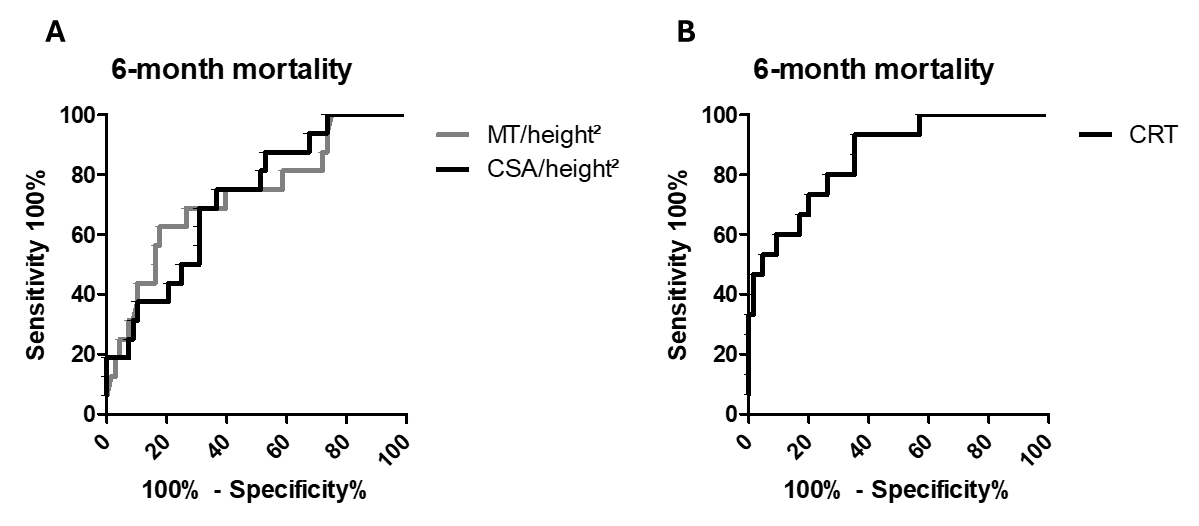
Suppl. Fig. 6**: (**A**) ROC of CSA_RFM_/height², MT_RFM_/height² for 6-month mortality. (**B**) ROC of CRT for 6-month mortality

**
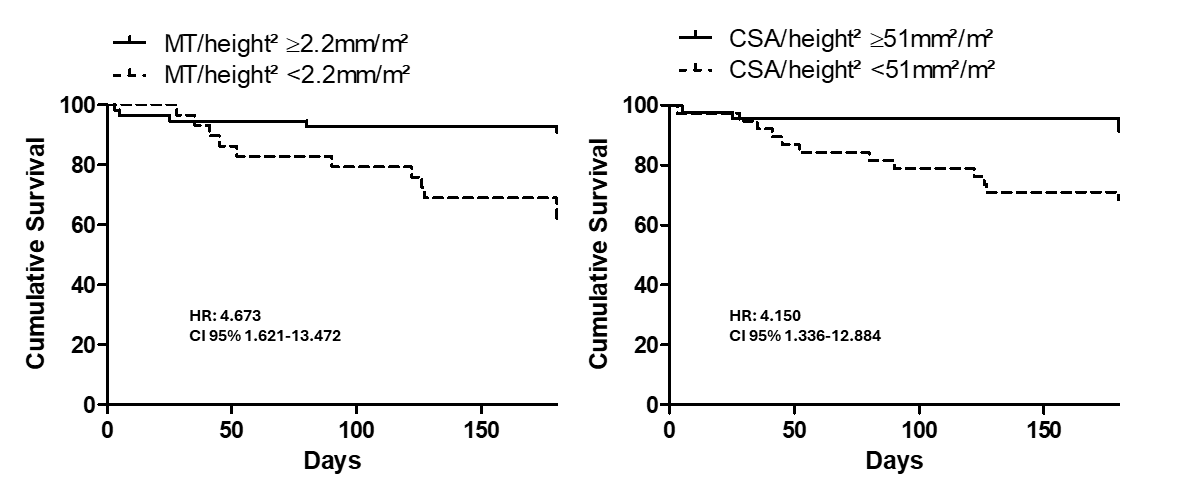
**

**Suppl. Fig. 7**: Survival of patients classified by MT/height² and CSA/height² of RFM as analysed using Kaplan-Meier analysis.

**
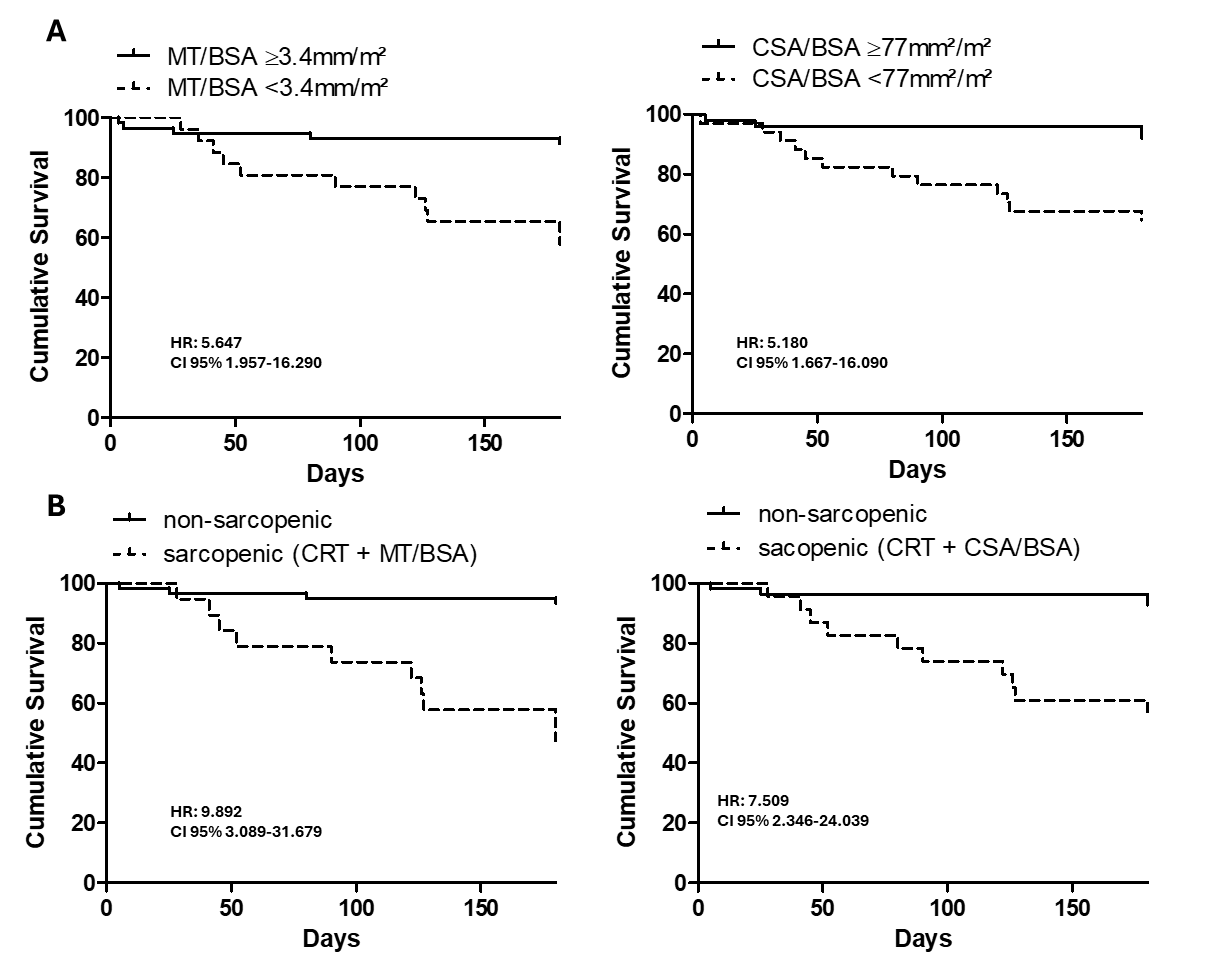
**

**Suppl. Fig. 8**: (**A**) Survival of patients classified by MT/BSA and CSA/BSA of RFM as analysed using Kaplan-Meier analysis. (**B**) Survival analysis of patients classified as ‘sarcopenic’ according to CRT >15s and MT/BSA <3.4mm/m² and CRT >15s and CSA/BSA <77mm²/m².


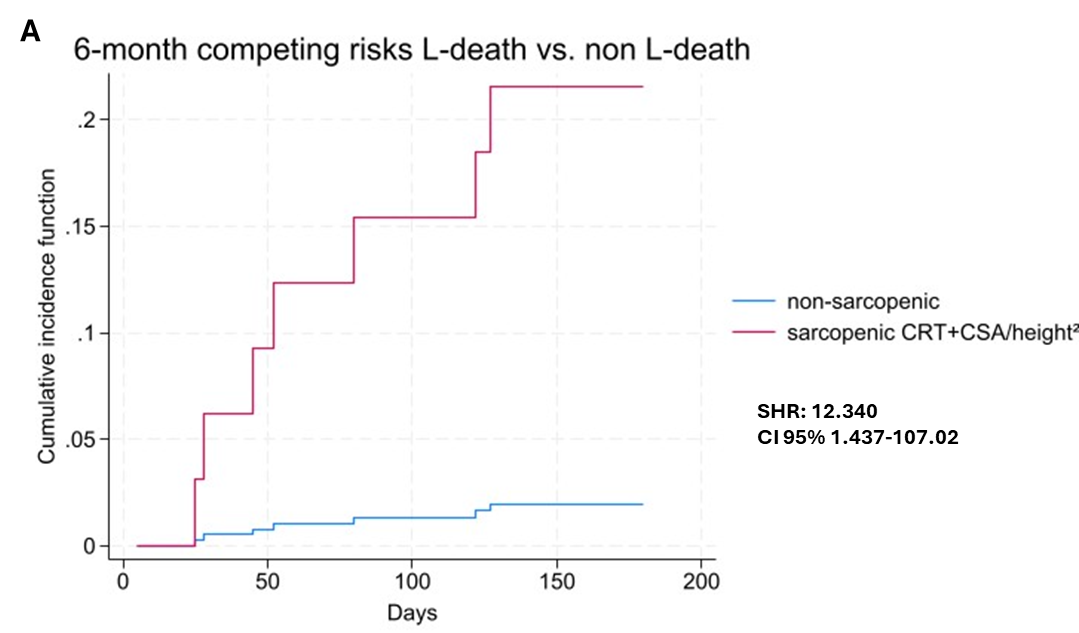


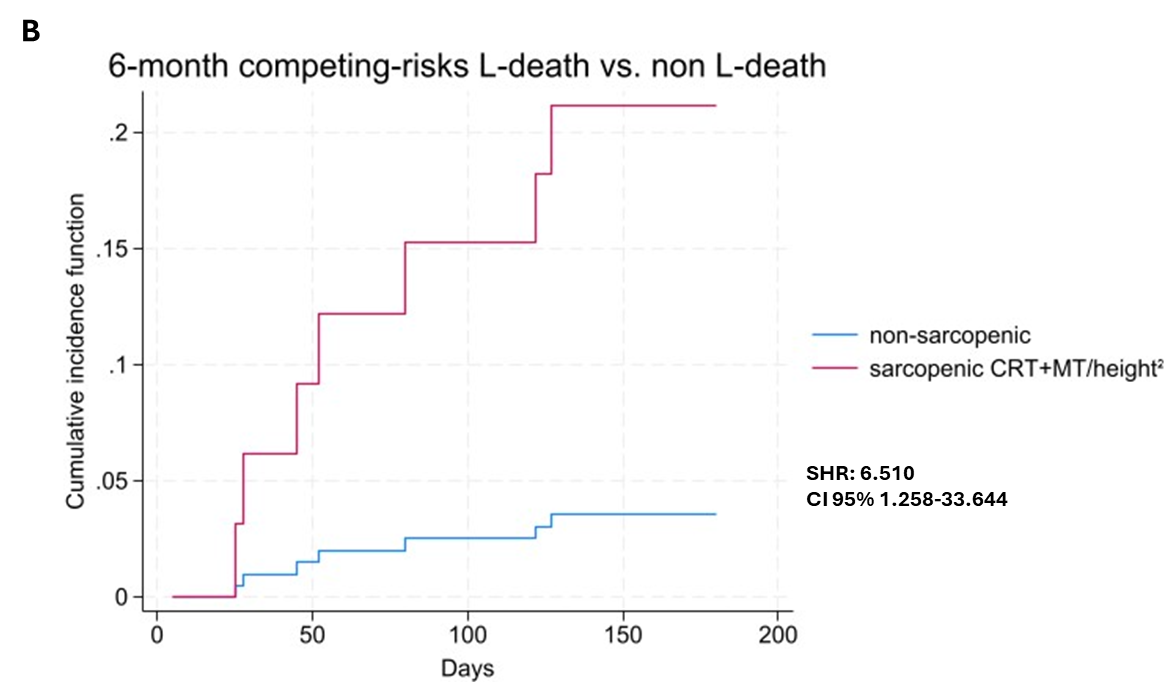


**Suppl. Fig. 9**: The 6-month cumulative incidence function of liver-related death (L-death) with non liver-related death (non L-death) as competing event was significantly higher in sarcopenic patients defined by CRT >15s and MT/height² <2.2mm/m² (**Panel A**) as well as CRT >15s and CSA/height² <51mm²/m² (**Panel B**).


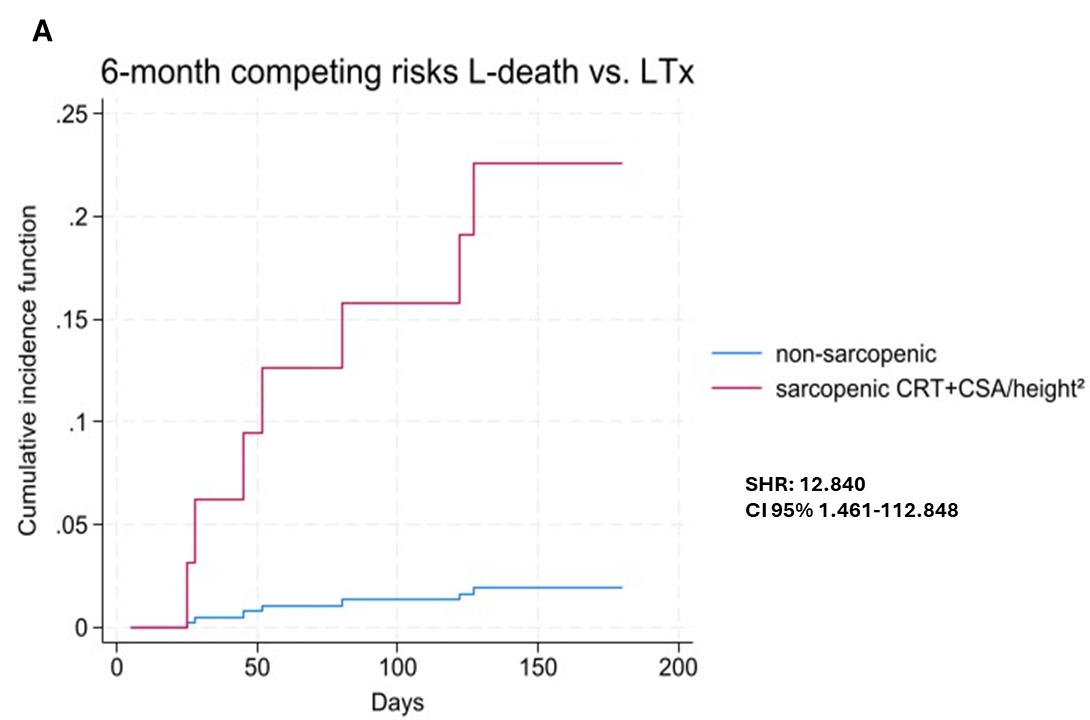


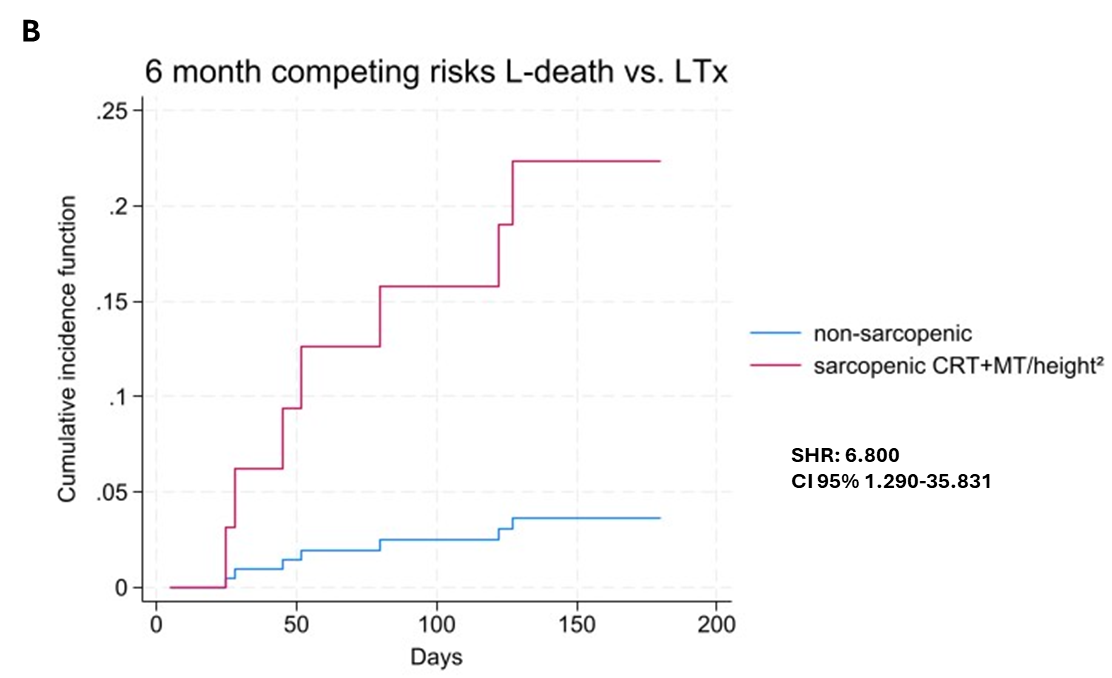


**Suppl. Fig. 10**: The 6-month cumulative incidence function of liver-related death (L-death) with liver transplantation (LTx) as competing event was significantly higher in sarcopenic patients defined by CRT >15s and MT/height² <2.2mm/m² (**Panel A**) as well as CRT >15s and CSA/height² <51mm²/m² (**Panel B**).
